# Anatomical landmarks for ultrasound‐guided rectus femoris diagnostic nerve block in post‐stroke spasticity

**DOI:** 10.1002/ajum.12354

**Published:** 2023-06-28

**Authors:** Salvatore Facciorusso, Stefania Spina, Giulio Gasperini, Alessandro Picelli, Mirko Filippetti, Franco Molteni, Andrea Santamato

**Affiliations:** ^1^ Villa Beretta Rehabilitation Center Valduce Hospital Costa Masnaga Lecco Italy; ^2^ Spasticity and Movement Disorders ‘ReSTaRt’ Unit, Physical Medicine and Rehabilitation Section, Policlinico Riuniti Hospital University of Foggia Foggia Italy; ^3^ Neuromotor and Cognitive Rehabilitation Research Center, Department of Neurosciences, Biomedicine and Movement Sciences University of Verona Verona Italy

**Keywords:** diagnostic nerve block, rectus femoris, spasticity, stiff knee gait

## Abstract

**Introduction/Purpose:**

To determine the location of the rectus femoris (RF) motor branch nerve, as well as its coordinates with reference to anatomical and ultrasound landmarks.

**Methods:**

Thirty chronic stroke patients with stiff knee gait (SKG) and RF hyperactivity were included. The motor nerve branch to the RF muscle was identified medially to the vertical line from anterior superior iliac spine and the midpoint of the superior margin of the patella (line AP) and vertically to the horizontal line from the femoral pulse and its intersection point with the line AP (line F). The point of the motor branch (M) was located with ultrasound, and nerve depth and subcutaneous tissue thickness (ST) were calculated.

**Results:**

The coordinates of the motor branch to the RF were 2.82 (0.47) cm medially to the line AP and 4.61 (0.83) cm vertically to the line F. Nerve depth and subcutaneous tissue thickness were 2.71 (0.62) cm and 1.12 (0.75) cm, respectively.

**Conclusion:**

The use of specific coordinates may increase clinicians' confidence when performing RF motor nerve block. This could lead to better decision‐making when assessing SKG in chronic stroke patients.

## Introduction

Spasticity is a velocity and muscle length‐dependent increase in resistance to the externally imposed muscle stretch.^1^ It shares similar pathophysiological origins with abnormal synergies, inappropriate muscle activation and anomalous muscle co‐activation.^2^

Spasticity is mainly a neurogenic phenomenon that results in unopposed excitatory descending inputs to spinal stretch reflex circuits.^3^ Lower limb muscle spasticity may alter postural stability and ambulation.^4^ Common patterns that affect gait include spastic equinovarus foot and stiff knee. Although there is a general consensus that plantar flexor spasticity contributes to equinovarus pattern,^5^ the role of the rectus femoris (RF) and other knee extensor muscles as contributors to stiff knee gait (SKG) is not well defined.

Stiff knee gait is a gait disturbance frequently associated with spastic post‐stroke hemiparesis. It may lead to an inefficient gait pattern and increased energy cost of walking.^6^ Stiff knee gait is characterised by limited knee flexion during the swing phase, and hyperactivity of the RF is thought to be a potential contributing factor. Common therapies for RF spasticity include injection with botulinum neurotoxin into the muscle, physical therapy and surgical strategies such as selective neurotomy. Some studies demonstrated improvements in knee flexion during swing phase after chemodenervation.^7^ However, many authors have emphasised that factors other than RF spasticity may contribute to SKG, such as insufficient ankle push‐off strength^8^ and hip pull‐off.^9^ A diagnostic motor nerve block (DNB) is advised to identify the extent of RF spasticity's contribution to SKG and predict clinical outcomes.^10^

Diagnostic motor nerve block consists of an anaesthetic injection near to the peripheral nerve branch in order to temporarily reduce nervous conduction.^11^ In rehabilitation settings, DNBs are commonly used to differentiate muscle contracture from muscle hyperactivity in patients living with post‐stroke spasticity and other spasticity‐related neurological disorders.^12^ With regard to SKG, DNBs may be used to determine the role of the overactive RF in this pathological pattern. Anaesthetic solution has to be administered close enough to the target nerves to achieve prompt and thorough interruption of nerve conduction. Various methods have been used to guide needle placement, including anatomic surface landmarks, identification of neighbouring bony and vascular structures, or electrical stimulation. In addition, nerve imaging by ultrasound has recently become available as a non‐invasive and time‐efficient tool that can accurately identify soft tissue and vascular nerve bundles. Indeed, during the ultrasound‐guided DNB procedure, identifying vessels with the estimated needle trajectory can help avoid inadvertent vascular puncture and related complications.

The anatomical description of motor nerve branches of quadriceps muscle was previously described in a cadaveric study.^13^ However, to the best of our knowledge, there are no studies investigating ultrasound characteristics and landmarks to target motor nerve branch to the RF in stroke population. The aim of this study was to identify anatomical and ultrasound landmarks of the femoral motor nerve branch to the RF to help clinicians during the motor nerve block procedure in stroke patients.

## Methods

A single‐centre observational study was conducted from January 2022 to June 2022. All participants (aged 18 and older) were outpatients with a diagnosis of chronic stroke (duration since event >6 months) and spastic quadriceps with a Modified Ashworth Scale (MAS) score ≥1. No subjects had previously received botulinum toxin type‐A (BoNT‐A) injection for the quadriceps spasticity. Patients having fixed contractures (score 4 on the MAS) or skeletal deformities, previous surgical or neurotomy of the afflicted lower limb, and intrathecal pharmacological treatment were also excluded from the study (*i.e*. baclofen). All participants were outpatients scheduled to receive selective DNB of the femoral motor nerve branch to the RF muscle. All subjects with a Functional Ambulation Classification (FAC) ≥2 were able to ambulate. In accordance with the Declaration of Helsinki, written informed consent for participation in the study was obtained and verified by the local ethics committee and competent authority.

A RS80A ultrasound system (Samsung Medison Samsung Medison Co., Ltd., Seoul, Korea) with a 3.0‐ to 12.0‐MHz linear array ultrasound transducer ‘L3‐12A’ was used to perform real‐time B‐mode ultrasonography on all patients. All patients had the same settings. The depth was set to 42 mm, and the focus zone was manually placed based on the RF motor branch depth. The ultrasound measurements of nerve depth (ND) and subcutaneous tissue thickness (ST) were taken using the ultrasound unit.

A Stimuplex HNS 12 peripheral nerve stimulator (B. Braun, Melsungen, Germany) with disposable pre‐gelled silver or silver chloride surface electrodes was used.

### Procedure

The patient was placed in the supine position. The ipsilateral extremity was abducted 10–20 degrees and slightly externally rotated with the lateral edge of the foot resting on the table.

First, the following landmarks were individualised and marked on patients:

- Point F: femoral arterial pulse at inguinal ligament;
- Point A: anterior superior iliac spine (ASIS);
- Point P: midpoint of superior edge of patella;
- Line AP: line between ASIS and the superior edge of patella; and
- Line F: intersection line between femoral arterial pulse and the line AP.

**Figure 1:**
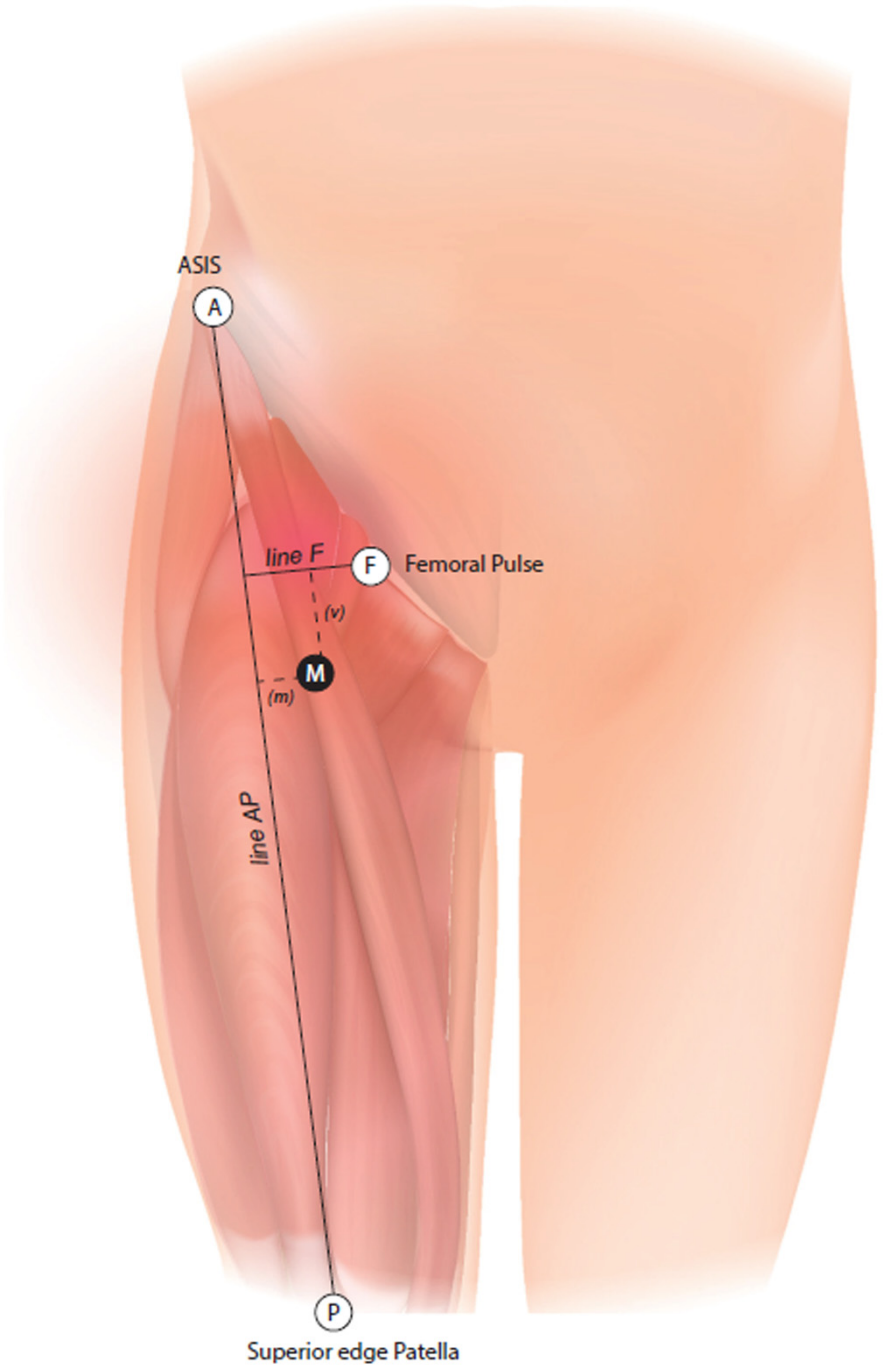
Anatomical landmark for RF motor nerve branch. Point A: ASIS anterior superior iliac spine; Point P: superior edge patella; Point F: femoral pulse at inguinal ligament; line AP; line F; Point M: motor branch reaches medial margin of RF muscle; m: medial distance; v: vertical distance.

Anatomical landmarks are represented in Figure 1.

The initial probe position (IPP) was placed in the axial plane just under the line F and 1 cm lateral to point F. From IPP, we moved laterally to the line AP. Distal to this scan, the femoral nerve splits into its motor branches. Ultrasonographic landmarks medially to laterally were as follows: femoral vein, femoral artery, femoral nerve and sartorius muscle. We tracked the motor nerve branch to the RF muscle that appears on the inferomedial border of the RF aponeurosis, and point M was determined and marked.

**Figure 2:**
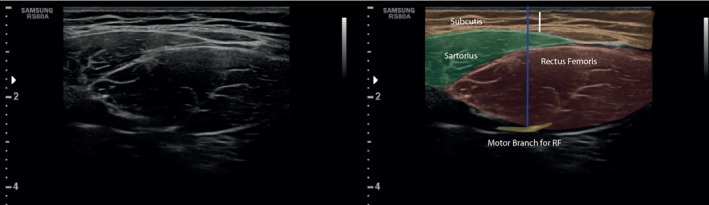
Ultrasound scan and schematic representation of the superior thigh muscle in its short axis. Blue line: nerve depth; white line: subcutaneous tissue; RF: rectus femoris.

The motor branch can be seen as a hyperechoic structure in its longitudinal view in continuity with the main trunk (Figure 2). Soon after, arborisations with sub‐branches piercing the RF should be identified.

The correct position was verified by electrostimulation of the motor branch. An electrified needle was inserted in plane with 30° inclination. Stimulation intensity was considered acceptable when muscle twitch appears at 0.5 mA, 0.1 ms and 2 Hz. The point M was confirmed and marked. A millimetric calliper was used to determine the following:

- medial distance (m) from the line AP to the point M and
- vertical distance (v) from the line F to the point M.

During the scanning procedure, the ND and the subcutaneous tissue thickness (ST) were calculated. The ND was measured as the distance between the upper perineurium of the FN and the line separating the dermis from fat; the ST as the distance between the line separating the derma from fat and the upper aponeurosis of the first muscle layer above the RF. All evaluations were made by a single, well‐trained operator with 7 years of experience in performing ultrasound‐guided procedures. To minimise the potential variability in ND measurement due to operator pressure, the probe was placed gently on the skin surface.

For DNB, we injected 2 mL of lidocaine 2.0% within the epineurium of the motor branch at currents between 0.3 and 0.5 mA. Lidocaine is the anaesthetic of choice for this procedure due to its short onset time (about 10 min) and mean duration of almost 2 h.

### Clinical measures

The MAS was used to evaluate spastic quadriceps tone. It is a 6‐point scale grading with respect to the muscle tone: 0—no increase; 1—slight increase at the end of the range of motion; 1+—slight increase through less than half of the range of motion; 2—more marked increase through most of the range of motion; 3—considerable increase; and 4—joint is rigid.^3^ The FAC is a 6‐point scale that was used to measure the need for support during ambulation from inability (score 0), physical assistance with continuous contact from one person (score 1), physical assistance with intermittent contact from one person (score 1), verbal supervision without physical contact (score 3), independence in walking with some help on stairs or uneven surfaces (score 4) and independence in walking anywhere (score 5).^14^

### Statistical analysis

To assess all variables, we used a descriptive statistic. The mean and standard deviation were used to report quantitative variables. The median was used to report ordinal variables. Only for statistical purposes was the MAS score considered from 0 to 5 (rather than 0 to 4), where 2 was intended as 1+ of the original scale. The Shapiro–Wilk test was used to determine whether the distribution was normal. The association between sonographic measures and demographic variables was investigated using a Spearman's rho correlation test. The Statistical Package for Social Sciences was used to conduct the statistical analysis (IBM SPSS Inc., Armonk, NY, USA). The significance threshold was set to P ≤ 0.05.

## Results

A total of 30 chronic stroke patients were enrolled. The demographic and clinical characteristics are presented in Table 1.

**Table 1:** Demographic and clinical features.

Characteristics	
Age (years), mean (SD)	59.25 (11.28)
Gender (male/female)	12/18
Time since event (years), mean (SD)	2.31 (2.28)
Side affected (right/left)	13/17
Type of stroke (ischaemic/haemorrhagic)	17/13
BMI (kg/m^2^)_,_ mean (SD)	27.67 (3.55)
Knee extensors spasticity (MAS) Median (min–max)	2 (1–3)
FAC median (min–max)	3 (2–4)

BMI, body mass index; FAC, Functional Ambulation Classification; MAS, Modified Ashworth Scale; SD, standard deviation.

The RF nerve at point M was located in all subjects. The mean distance between ASIS and point M was 10.21 (1.73) cm.

The anatomical landmark coordinates of point M are as follows:

- Medial distance: 2.82 (0.47) cm.
- Vertical distance: 4.61 (0.83) cm.

The ultrasonographic measures at point M are as follows:

- Subcutaneous tissue: 1.12 (0.75) cm.
- Nerve depth: 2.71 (0.62) cm.

Ultrasound image of the RF of the first motor nerve branch to the RF muscle is shown in Figure 2.

No significant correlations were found between ultrasound characteristics and demographic and clinical measures (P > 0.05), nor were any adverse events reported after injection in any patient.

## Discussion

This study provides a reliable procedure to improve the accuracy of DNB by providing anatomical landmarks and ultrasound characteristics of the first motor branch to RF. We decided to study RF as we consider the RF an important determinant in post‐stroke gait.

Gait problems are common in stroke patients: while approximately 85% of hemiplegic stroke patients regain walking ability, their gait patterns typically differ from those of healthy individuals. These differences negatively impact on biomechanics, overall body function and quality of life, and increase the risk of falls and functional dependency. Gait rehabilitation for stroke survivors aims to improve walking safety and speed, which are crucial for preventing falls and improving quality of life.^15^

Clinically, a wide range of gait abnormalities are observed, depending on the severity of weakness and spasticity, as well as the altered neural control. These gait impairments result from interactions between muscle weakness, spasticity and spastic synergistic patterns. The abnormal activation of RF muscle could lead to SKG, decreased speed gait and stability, which increase the risk of falling.^16^, ^17^ There is an increasing debate about the RF spasticity as a possible cause of SKG.^18^ Accordingly, the clinical decision‐making process to assess the involvement of the RF in the SKG requires a better understanding of its physiopathology and aetiology to increase the appropriateness of therapeutic protocols.

Goldberg *et al*.^19^ showed that increased forces of vasti and RF decrease the peak knee flexion velocity during double limb support, while RF overactivity alone is involved in the pre‐swing and swing phases. A recent paper confirms an association between quadriceps hyperreflexia and diminished knee flexion during the swing phase in SKG.^20^ Merlo *et al*.^18^ proposed a three‐dimensional gait analysis and dynamic electromyography to accurately evaluate and differentiate the causes of a SKG. These methods are time‐consuming and require multidisciplinary trained professionals and adequate instrumentation that is not available in all clinical settings. Moreover, the Duncan–Ely test, a clinical procedure for evaluating RF spasticity by passively flexing the knee quickly while the patient is lying prone and relaxed,^21^ has been shown to have little diagnostic utility for predicting aberrant RF activity in stroke survivors with SKG.^22^ In fact, a clinical test carried out on a bench in a static and relaxed position has limited diagnostic utility for predicting aberrant RF activity in stroke survivors with SKG. Instead, a motor branch block is recommended as it allows for temporary analysis of the patient's walking pattern without the action of the spastic muscle. Therefore, a diagnostic block was suggested as a useful tool to temporarily analyse the gait pattern of the patient and to study SKG^10^ before attempting a more prolonged therapy, such as botulinum toxin injection, or a more definitive therapy, such as neurotomy, fractional lengthening or tenotomy.

Botulinum toxin type‐A injection is frequently used as medical intervention to target the inappropriate activity of RF for gait improvement. Botulinum toxin type‐A acts at the neuromuscular junction by preventing the release of neurotransmitters leading to a ‘voluntary, reversible and transient’ paresis of the injected muscle. Several uncontrolled studies were conducted evaluating the effect of BoNT‐A treatment: BoNT‐A injected into RF has been shown to increase peak knee flexion by 5 or 8 degrees during swing, to improve knee angular velocity at toe‐off, to increase gait speed, to reduce the energy cost of walking and to improve coordination between hemiparetic thigh and shank.^23^, ^24^, ^25^, ^26^

In a meta‐analysis including nine articles about the effects of chemodenervation (neuromuscular block and motor branch block) of the RF on SKG, Tenniglo *et al*.^7^ showed a significant improvement in peak knee flexion during swing, while it was unclear whether chemodenervation improves walking speed and energy cost. Conversely, another study demonstrated that the neurotomy of the femoral motor branch to the RF improves kinetics, kinematics and walking parameters at 3 months.^27^

The RF is innervated by the femoral nerve. The femoral nerve is the largest branch of the lumbar plexus and is made up of the dorsal divisions of the anterior primary rami of spinal nerves L2, L3 and L4. The femoral nerve enters the femoral triangle just lateral to the femoral artery, travelling behind the inguinal ligament. The nerve divides into anterior and posterior divisions below the inguinal ligament. The sartorius is supplied by the anterior division, which also gives rise to two cutaneous branches: the intermediate and medial cutaneous nerves of the thigh. The RF, three vasti and articularis genu are supplied by the posterior division, which has only one cutaneous branch.^28^ Plante *et al*. identified not more than four branches in the RF (two branches in 69.9% of cases). They found that the average distance between the anterior–inferior iliac spine and the first ramification of the femoral nerve to the RF was 8.6 (1.4) cm. The distance from other branches is not specified.^29^ In our study, we found a mean distance of 10.21 (1.73) cm from ASIS to first motor branch, and this result is in line with that of a previous study.^13^

Sung *et al*. in a sample of 31 patients used a DNB with lidocaine prior to treating 19 patients with a phenolic motor branch block of the RF to predict the outcome. They demonstrated significant improvements in gait speed maximal knee flexion angle at swing phase. For the motor nerve block, they used an electrical stimulation‐guided nerve block with anatomical landmarks derived from a previous experience of anatomic dissection of 12 cadavers. Despite their anatomical knowledge, in their sample, three out of 19 treated patients complained of adverse effects mainly due to the unwanted block of other femoral nerve branches.^30^

In our study, patients were evaluated using ultrasound, a routine imaging technique that guides anaesthetic or any other chemical agent injection and also allows nerve blocks to be performed by targeting the location of the motor nerve branches.^11^ The use of ultrasound is not only an essential skill for performing an ultrasound‐guided nerve block but also enables non‐invasive tissue structure imaging. Furthermore, we hypothesised that ultrasound imaging of the RF motor branch could limit risk related to an inaccurate injection, as it allows a good visualisation of a precise point where the motor branch reaches the inferomedial border of the RF.

In a recent study, Picelli *et al*.^31^ identified the anatomical landmarks of tibial motor nerve branches to the gastrocnemii, soleus and tibialis posterior muscles. They found that ultrasound‐guided selective motor nerve blocks may be useful to localise motor nerve branches in the management of spastic equinovarus foot. They suggested that ultrasound should be coupled with needle electrical stimulation in order to maximise precise identification of motor nerve branches.^31^ Electrical stimulation may improve the safety of nerve block procedure by avoiding nerve injury mainly due to unwanted intraneural injection. The location of the RF motor nerve branch has been determined previously by Sung *et al*.^32^ in 22 adult cadavers. These landmarks are often used by other authors.^24^ Our findings are consistent with those of Sung *et al*. However, our study has two additional strengths. First, we examined live patients in a real clinical setting, rather than cadaveric models. While cadavers may provide a viable training option, they have limitations such as muscle rigidity, limited flexibility and altered tissue. Second, we chose anatomical landmarks that are easy and rapid to identify in any patient in clinical practice. Our study found that the line AP and the line F are very close to point M with a medial distance of 2.82 (0.47) cm and a vertical distance of 4.61 (0.83) cm. This reduces the exploration field for correct nerve location. In patients with a very large abdomen, lateral decubitus may help to palpate ASIS more easily. Once the point M was found, we evaluated the correct location of the motor nerve branch using ultrasound and an in‐plane technique for needle injection to visualise the tip of the needle. We do not recommend an out‐of‐plane technique as it may reduce the accuracy of needle placement. The ‘elevator technique’ with ultrasound probe, which consists of tracking it proximally and distally, may be used to confirm proper needle placement.^33^ In addition, in some cases, we suggest to verify anatomical landmarks for the femoral artery at inguinal ligament with ultrasound.

Our study found that ST at point M is relatively thin, with a measurement of 1.12 (0.75) cm, which is consistent with another study on healthy subjects.^34^ Variability in ST may be a limit in many ultrasound‐guided procedures as it may increase the difficulty of visualising the target and decrease the precise location. However, our study suggests that there is little difference in ST depth and motor branch location among subjects. Our study suggests that the consistency and repeatability of the measures we suggested are good, as indicated by the little difference in ST depth and motor branch location among subjects. Additionally, the ND is relatively small at 2.71 (0.62) cm, which allows for simpler nerve location with ultrasound imaging. We found no significant differences between ultrasound characteristics and demographic or clinical measures.

This study has some important limitations. First, we only identified the first motor nerve branch for RF muscle. However, once the first branch is targeted following the suggested landmarks, other branches could be visualised, stimulated and blocked slightly down. Second, although we only found small differences in anatomical landmarks among patients, a larger sample size should be necessary to confirm these data. Lastly, our sample consisted of adult stroke patients and may not be generalisable among other populations. Future studies could verify gait improvement with an instrumental gait analysis and muscle activation by means of dynamic electromyography.

## Conclusion

To conclude, ultrasound‐guided diagnostic nerve block represents a reliable and safe method to localise the motor nerve branch to the RF motor branch for evaluating and verifying the RF contribution in SKG. In a daily clinical setting, the anatomical landmarks proposed in this study may help clinicians to improve precise identification by also coupling ultrasound with needle electrical stimulation. Furthermore, clinicians without access to ultrasound may benefit from these simple anatomical and ultrasonographic landmarks to improve the accuracy of the DNB procedure on the motor branch to the RF.

## Author contributions

**Salvatore Facciorusso:** Conceptualization (lead); methodology (lead); data curation (lead); investigation (lead); writing – original draft (lead); writing – review and editing (lead). **Stefania Spina:** Conceptualization (lead); methodology (lead); writing – original draft (lead); writing – review and editing (lead). **Giulio Gasperini:** Conceptualization (equal); investigation (lead); Writing – review and editing (equal). **Alessandro Picelli:** Conceptualization (equal); methodology (equal); writing – review and editing (lead). **Mirko Filippetti:** Conceptualization (equal); methodology (equal); writing – review and editing (lead). **Franco Molteni:** Conceptualization (equal); methodology (equal); writing – review and editing (lead). **Andrea Santamato:** Conceptualization (equal); methodology (equal); writing – review and editing (lead).
